# Short-term cyclical stretch phosphorylates p38 and ERK1/2 MAPKs in cultured fibroblasts from the hearts of rainbow trout, *Oncorhynchus mykiss*

**DOI:** 10.1242/bio.049296

**Published:** 2020-01-10

**Authors:** Elizabeth F. Johnston, Todd E. Gillis

**Affiliations:** Department of Integrative Biology, University of Guelph, Ontario, Canada, N1G 2W1

**Keywords:** Cardiac fibroblasts, Cell signaling, Mechanotransduction, Protein phosphorylation

## Abstract

The form and function of the rainbow trout heart can remodel in response to various stressors including changes in environmental temperature and anemia. Previous studies have hypothesized that changes in biomechanical forces experienced by the trout myocardium as result of such physiological stressors could play a role in triggering the remodeling response. However, there has been no work examining the influence of biomechanical forces on the trout myocardium or of the cellular signals that would translate such a stimuli into a biological response. In this study, we test the hypothesis that the application of biomechanical forces to trout cardiac fibroblasts activate the cell signaling pathways associated with cardiac remodeling. This was done by cyclically stretching cardiac fibroblasts to 10% equibiaxial deformation at 0.33 Hz and quantifying the activation of the p38-JNK-ERK mitogen activated protein kinase (MAPK) pathway. After 20 min, p38 MAPK phosphorylation was elevated by 4.2-fold compared to control cells (*P*<0.05) and after 24 h of stretch, p38 MAPK phosphorylation remained elevated and extracellular-regulated kinase 1/2 was phosphorylated by 2.4-fold compared to control (*P*<0.05). Together, these results indicate that mechanotransductive pathways are active in cardiac fibroblasts, and lead to the activation of cell signaling pathways involved in cardiac remodeling.

## Abstract (precis)

**Summary:** Here we demonstrate that 10% deformation at 0.33 Hz of trout cardiac fibroblasts stimulates p38-JNK-ERK mitogen activated protein kinase pathways. This suggests that mechanotransductive pathways trigger cardiac remodeling in trout.

## INTRODUCTION

The fish heart has a significant capacity to remodel in response to changes in physiological conditions (Alderman et al., 2012; Johnson et al., 2014; Keen et al., 2016; Klaiman et al., 2011; Marques et al., 2008; Simonot and Farrell, 2007). This remodeling can involve concurrent changes to the active (muscle) and passive (extracellular matrix) components of the myocardium (Keen et al., 2017). For example, cold acclimation causes an increase in deposited collagen in the extracellular matrix (ECM) of the rainbow trout myocardium (Keen et al., 2016; Klaiman et al., 2011). This is interesting because a significant increase in collagen deposition in the mammalian heart is associated with a pathological condition that is permanent and results in diastolic dysfunction (Burchfield et al., 2013; Zeisberg et al., 2007). Comparatively, the increase in collagen content in the trout heart with cold acclimation is associated with an increase in function, and is reversible (Keen et al., 2016, 2017; Klaiman et al., 2011).

Recent studies, working to identify the cellular signals responsible for regulating collagen deposition in the trout heart, have demonstrated that treatment of cultured trout ventricular fibroblasts with transforming growth factor-beta 1 (TGF-β1) induces collagen synthesis and deposition into the extracellular matrix (Johnston and Gillis, 2017, 2018), and that this cytokine causes changes in the transcript levels of collagen type 1 (col1), matrix metalloproteinase 2 (MMP2), and tissue inhibitor of metalloproteinase 1 (TIMP 1), which would support the observed increase in collagen. In addition, work by Johnston and Gillis (2018) demonstrated that TGF-β1 treatment of trout cardiac fibroblasts caused an increase in the phosphorylation of small mothers against decapentaplegic 2 (Smad2), a cell signaling molecule involved in regulating collagen deposition in the ECM of mammalian cardiac fibroblasts (Lee et al., 2002; Nakao et al., 1997; Nyati, 2012). In mammalian heart models, increased levels of stretch and/or shear stress on the endocardium triggers TGF-β1 production (Katsumi et al., 2004; MacKenna et al., 1998) and this cytokine then stimulates cardiac fibroblasts to increase the transcription of *timp-2* (Visse and Nagase, 2003) and collagen type I (*col1*; Kolosova et al., 2011; Reed, 1994), resulting in an increase in connective tissue in the ECM. One demonstrated cause of this response is an increase in blood viscosity, as this leads to an increase in cardiac workload, and as a result increased cellular deformation, thereby activating mechanically sensitive cellular proteins that then trigger the responsible signaling pathways (Husse et al., 2007; Reed et al., 2014; Waring et al., 2014).

Graham and Farrell (1989) have demonstrated that cold acclimation of trout causes an increase in blood viscosity, and suggest that this could be the trigger for cold-induced cardiac hypertrophy. An increase in blood viscosity increases vascular resistance and, therefore, the amount of work performed by the heart (Farrell, 1984; Keen et al., 2017). As discussed above, such changes cause increased cellular deformation and can activate stretch-sensitive signaling pathways (Husse et al., 2007; Reed et al., 2014; Waring et al., 2014). It is these pathways that could induce cardiac remodeling in these fish. Related to this, Keen et al. (2018) have demonstrated that cold acclimation of trout influences the transcript levels of the different isoforms of matrix metalloproteinase and collagen in the trout heart and suggest that these changes would support an increase in collagen deposition in the ventricle.

In this study, we tested the hypothesis that physiologically relevant levels of mechanical stretch of trout cardiac fibroblasts would stimulate the activation of the p38-JNK-ERK mitogen activated protein kinase (MAPK) pathway. This signaling pathway is involved in the pathological remodeling of the mammalian heart (Chiquet et al., 2009), is triggered by mechanical cues, and is activated by the phosphorylation of the associated MAPKs, including p38 and ERK1/2 (Lal et al., 2007; Verma et al., 2011). We predicted that exposure of trout cardiac fibroblasts to physiologically relevant levels of stretch would result in the activation of the p38-JNK-ERK MAPK pathway and would be detected by the increased phosphorylation of these proteins.

## RESULTS AND DISCUSSION

### Initiation of MAPK signaling

**Fig. 1.**
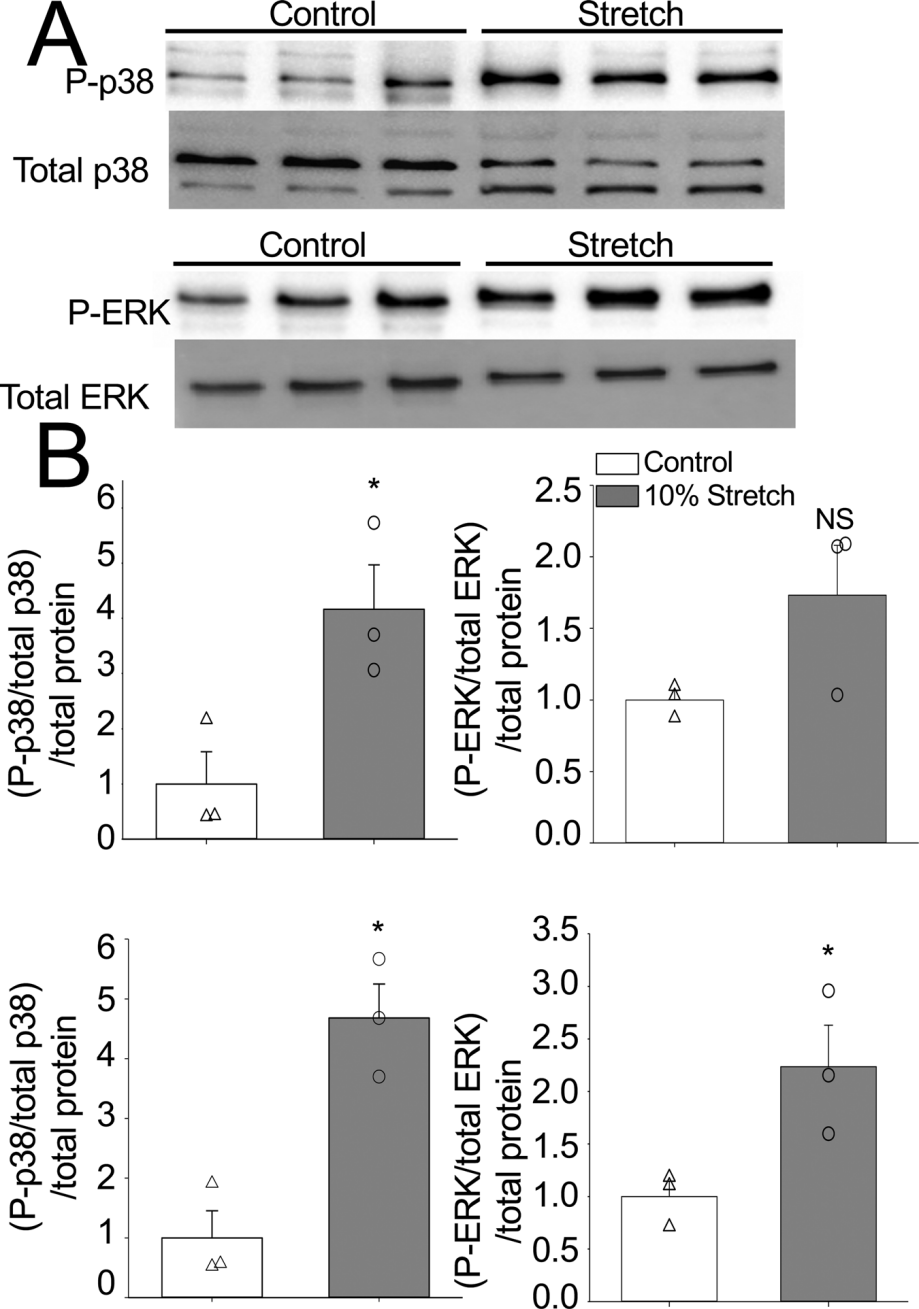
**Activation of p38 and ERK1/2 pathways in response to stretch.** (A) Representative western blot images of phosphorylated p38 (top) and ERK1/2 (bottom) after 24 h of stretch (see Fig. S1 for remaining blot images). (B) Phosphorylation levels of p38 and ERK proteins in stretched and control (unstretched) cells were first normalized to total p38 and total ERK. These values were then normalized to total protein within the samples and control values were set to 1. Phosphorylation levels between stretched and control cells were compared using a two-tailed *t*-test. Asterisks (*) indicate a significant effect of stretch on MAPK phosphorylation (*P*<0.05). Open triangles (Δ) signify individual control (unstretched) data points, and open circles (○) are individual data points from stretched cells. Points with similar numerical values were staggered for better readability. Error bars represent standard error (s.e.) of the mean of each group. 20 min p38 control, s.e.=±0.58; stretched, s.e.=±0.8. 20 min pERK control, s.e.=±0.07; stretched, s.e.=±0.35. 24 h p38 control s.e.=±0.45; stretched s.e.=±0.57. 24 h pERK control=±0.15; stretched s.e.=±0.39. *N*=3 for each group. The *n*-value represents a fibroblast line from the same individual fish maintained in separate passages, cryopreserved on a different passage number and day, and thawed for experiments on different days.

The activation of MAPKs through mechanosensitive components involves mediation of the originating extracellular signal through small G proteins such as Ras or Rho (Rajalingam et al., 2007). When Ras is activated via phosphorylation, it is able to phosphorylate downstream targets, such as MAPKs (Molina and Adjei, 2006). In the mammalian heart, this leads to changes in gene expression and resultant protein expression that underpin the cellular responses associated with cardiac remodeling (Pramod and Shivakumar, 2014; Sinfield et al., 2013). In the current study, there was no difference in the levels of total p38 MAPK or ERK protein between control and the treatment timepoints (*P*>0.05); however, there was a 4.2-fold increase in p38 MAPK phosphorylation after 20 min of 10% equibiaxial deformation (Fig. 1). In addition, after 24 h hours of stretch, the higher level of p38 MAPK phosphorylation was maintained and the level of ERK phosphorylation was 2.4-fold that of control (*P*<0.05) (Fig. 1). This indicates that the trout fibroblasts respond rapidly to biomechanical stimulation and that the response is sustained for the duration of the applied stressor. It remains to be determined, however, which mechanosensitive cellular components initiated the signal transduction pathway. One likely candidate, and a target for future studies, are integrins. These proteins anchor the cytoskeleton to the extracellular matrix and are involved in ERK1/2 and p38 signaling in mammalian fibroblasts (Katsumi et al., 2004; Ross et al., 2013).

### Conservation of MAPK signaling

One of the established effects of the activation of the p38-JNK-ERK pathway in mammalian cardiac fibroblasts is the stimulation of transcriptional factors that lead to an increase in collagen deposition (Li et al., 2018; Molkentin et al., 2017; Travers et al., 2016; Pramod and Shivakumar, 2014; Sinfield et al., 2013). In addition, work by Husse et al. (2007), has demonstrated that cyclical stretch of rat cardiac fibroblasts results in an increase in the expression of collagen types I and III transcripts, mRNA's whose transcription is regulated by the p38-JNK-ERK pathway. Therefore, the increased phosphorylation levels of p38 and ERK1/2 in the trout cardiac fibroblasts suggests that the activation of the p38-JNK-ERK pathway in cardiac fibroblasts by mechanical stimulation is a conserved response within vertebrate hearts. The next study should determine if the p38-JNK-ERK pathway is activated in the trout heart during cold acclimation and how this affects collagen deposition. In addition, it is important to note that MAPKs are involved in a number of processes unrelated to remodeling, including cell growth and differentiation (Hommes et al., 2003; Whitmarsh, 2007). It is possible, therefore, that the activation of the p38-JNK-ERK pathway by biomechanical forces in the current study could also affect cellular pathways that are not directly associated with cardiac remodeling.

### Conclusions and perspectives

To the best of our knowledge, this is the first study to demonstrate that cultured fish cells are mechanotransductive, and that major intracellular pathways involved in remodeling in trout cardiac fibroblasts can be activated in response to stretch. The hypothesis that physical forces play a role in triggering the remodeling of the trout myocardium, as a result of cellular mechanotransduction, has existed for ∼30 years, but the direct effect of mechanical factors on remodeling cues in fish cells has not been investigated until now. The results of this study support this hypothesis and lend novel insight into the extracellular cues required to initiate the process of cardiac remodeling.

## MATERIALS AND METHODS

### Cell culture

Rainbow trout cardiac fibroblast cells were cultured and maintained as previously described (Johnston and Gillis, 2017). Cells between passages 19–21 were used in experiments outlined below. Cardiac fibroblasts were plated onto a collagen type I-coated BioFlex rubber membrane six-well plate at a density of ∼0.2-0.3×10^6^ cells ml^−1^ and allowed to attach for 7 days to ensure a sufficient matrix was established by the fibroblasts, so that they would not detach during the stretch experiments. Initially, cells were grown for 3 days prior to stretch experiments, but this resulted in loss of cellular adherence before 24 h.

### Stretch apparatus and application

A FlexCell FX-4000 apparatus (FlexCell International, Burlington, NC, USA) was used to stretch trout cardiac fibroblasts, with some modification. The baseplate vacuum inflow line was connected directly to a Leybold D8B rotary vane vacuum pump (Leybold, Cologne, Germany), and the outflow line was vented externally (atmospheric pressure). Vacuum pressure was controlled by a valve. Air inflow and outflow were regulated by solenoid valves, the current to which was controlled by a simple timer on a computer. By turning the power on to either solenoid valve, negative pressure could be applied alternately with atmospheric pressure, resulting in cyclical stretch of the rubber membranes. Percent deformation was estimated by placing an ink marking on a rubber membrane, and visualizing area deformation with a Zeiss SteREO Discovery. V12 dissection microscope, then analyzed in ImageJ. Cells were stretched cyclically at 10% deformation for 10 min, 20 min and 24 h at a frequency of 0.033 Hz. This magnitude of deformation was chosen based on previous studies examining stretch in cardiac fibroblasts of other species (Butt and Bishop, 1997; Guo et al., 2013). Furthermore, fish cardiac output modulation is achieved by changing stroke volume rather than frequency, and trout can modulate stroke volume by up to threefold, whereas mammals alter heart rate to maintain cardiac output (Keen et al., 2017; Shiels et al., 2006). It has been established that fish modulate ventricular volume to a much greater extent than mammals (Farrell, 1991; Shiels and White, 2008). Therefore, 10% stretch used in this study is within a physiologically relevant range and may actually represent a conservative approach for fish cells.

### Western blotting/phospho-blotting

Cells in the control and treatment groups were washed with 2 ml PBS, then 200 µl radioimmunoprecipitation assay (RIPA) buffer containing 1 µmol l^−1^ phenylmethane sulfonyl fluoride (PMSF, Sigma-Aldrich) and phosphatase inhibitors (50 mM sodium fluoride, 5 mM sodium pyrophosphate and 5 mM sodium orthovanadate), then incubated on wet ice for 5 min. Cells were removed from flasks by scraping and then transferred to a 1.5 ml Eppendorf tube and homogenized by being repeatedly flushed through a needle and syringe, on ice. The lysate was then maintained at 4°C with gentle agitation for 30 min, centrifuged for 20 min at 20,000* **g***, and the supernatant was reserved and measured for protein content using a bicinchoninic acid (BCA) assay (Bio-Rad, Hercules, USA). A 20 µg sample of protein, diluted in Laemmli buffer, was loaded into the lane of 12% polyacrylamide gel and run at 160 V for ∼1–1.5 h, at 4°C. Proteins were electro-blotted onto a polyvinylidene fluoride membrane with wet transfer at 30 V for 16 h at 4°C and blocked in 5% bovine serum albumin (BSA; Bioshop, Burlington, Canada) in Tris-buffered saline with 0.1% Tween 20 (TBST) (hereafter referred to as blocking buffer) for 1 h at room temperature, then incubated in 1:1000 anti-rabbit phospho-ERK1/2 or anti-rabbit phospho-p38 (Cell Signaling Technology, Danvers, MA, USA; catalogue numbers D13.13.4E and D3F9, respectively) in blocking buffer overnight at 4°C. The next day, blots were incubated in goat anti-rabbit IgG-HRP secondary antibody buffer (Santa Cruz Biotechnology, Dallas, USA) at a dilution of 1:1000 in blocking buffer. An Amersham ECL Plus detection kit (GE Healthcare, Chicago, USA) was used as per the manufacturer's instructions to induce chemiluminescence, which was subsequently visualized with the ChemiDoc MP imaging system (Bio-Rad) and then analyzed with the associated software. Blots were stripped for 7 min in mild membrane stripping buffer (0.2 M glycine, 0.1% SDS, 1% Tween20, pH 2.2), re-blocked, and then incubated in 1:1000 anti-rabbit total ERK1/2 or total p38 in blocking buffer (Cell Signaling Technology catalogue numbers D13E1 and 137F5, respectively). Phosphorylated p38 and ERK1/2 were first standardized to total p38 and total ERK1/2. This was done to ensure that changes in phosphorylation that were detected were not a result of differential levels of the MAPKs within a sample. These standardized data were then normalized to total protein from the same membranes using a SYPRO Ruby Blot Stain (Bio-Rad). The averages of phosphorylated MAPK/total MAPK/total protein were compared in statistical analyses outlined below.

### Statistical analysis

All data were initially subjected to a Shapiro–Wilk test for normality. Any data that failed to meet normality were log-transformed prior to analysis. Treatment and control data collected at the same time point were compared with a Student's *t*-test, with significance occurring at 95% confidence. The *n*-value represents a fibroblast line from the same individual fish maintained in separate passages, cryopreserved on a different passage number and day, and thawed for experiments on different days.

## Supplementary Material

Supplementary information
