# Supplementary material for: Short-term cyclical stretch phosphorylates p38 and ERK1/2 MAPKs in cultured fibroblasts from the hearts of rainbow trout, Oncorhynchus mykiss
Source: Biol Open. 2020 Jan 10;9(1):bio049296. doi: 10.1242/bio.049296 (PMC6994941; doi:10.1242/bio.049296)
Supplement: Supplementary information [file biolopen-9-049296-s1.pdf]

Biology Open (2019): doi:10.1242/bio.049296: Supplementary information

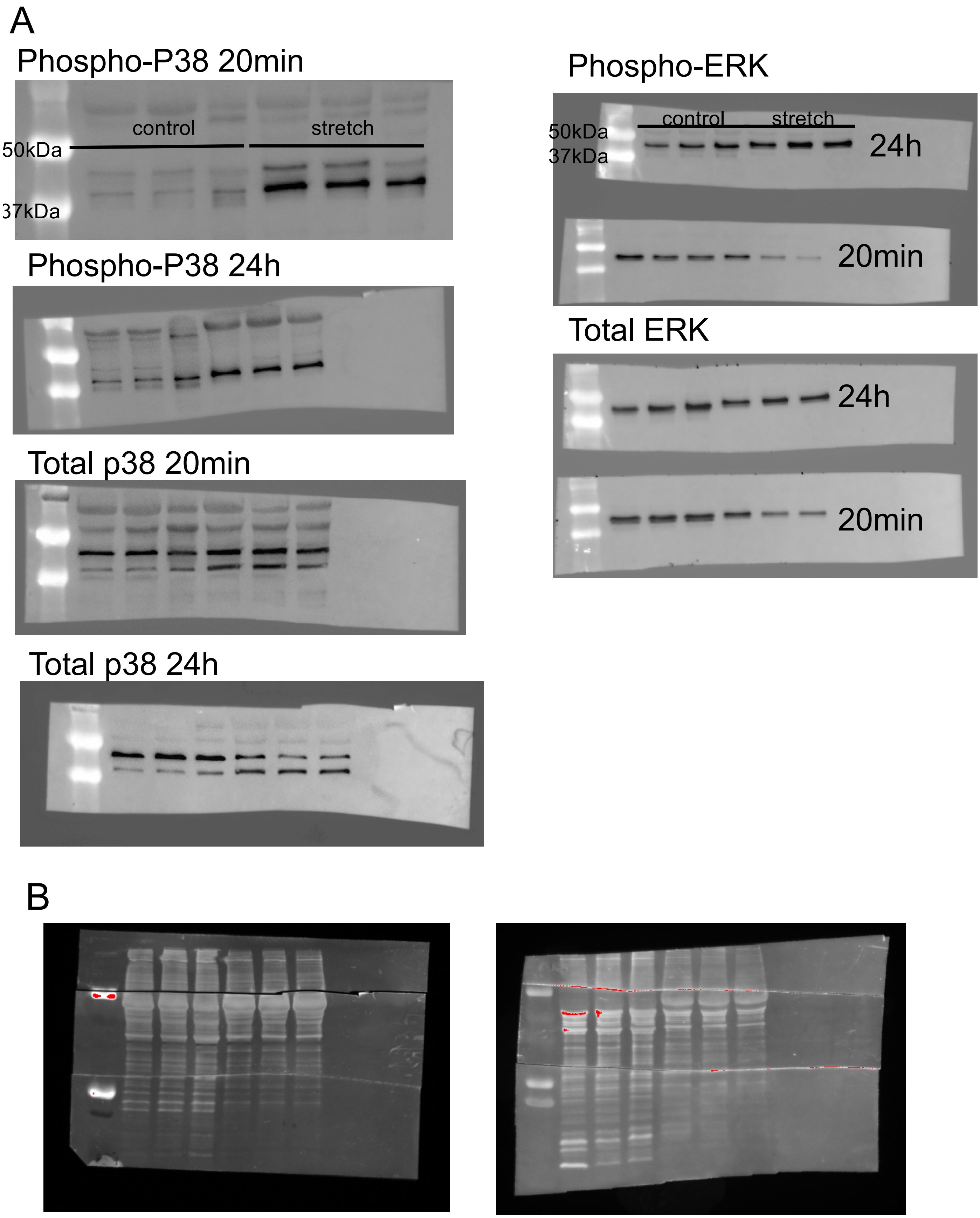

Figure S1. Western blot images not shown in fig. 1. A) Multichannel images of phosphorylated p38 and ERK1/2 Western blots. Antibody corresponding to MAPK examined is labeled for each image, and the type of sample ran in each lane is indicated above image. Ladder is in lane 1. Control samples were loaded into lanes 2-4. Stretched samples were in lanes 5-7. This configuration is the same for all blots. 37 and 50kDa bands shown are the same for all blots. B) Representative images of SYPRO Ruby total protein stain used for total protein quantification.

Biology Open • Supplementary information
